# Application of Laser Scanning to Assess the Roughness of the Diaphragm Wall for the Estimation of Earth Pressure

**DOI:** 10.3390/s21217275

**Published:** 2021-11-01

**Authors:** Marek Wyjadłowski, Zbigniew Muszyński, Paulina Kujawa

**Affiliations:** 1 Faculty of Civil Engineering, Wrocław University of Science and Technology, 50-370 Wrocław, Poland; marek.wyjadlowski@pwr.edu.pl; 2 Faculty of Geoengineering, Mining and Geology, Wrocław University of Science and Technology, 50-370 Wrocław, Poland; paulina.kujawa@pwr.edu.pl

**Keywords:** geodetic measurements, terrestrial laser scanning (TLS), surface topography measurement, earth pressure, 3D roughness parameters, diaphragm wall

## Abstract

The correct estimation of earth pressure is important for the design of earth retaining structures and depends, among others, on the surface morphology of retaining structures. The diaphragm wall created as a protection of a deep excavation located in an urbanized area was selected as a research object. Terrestrial Laser Scanning (TLS) was used for the investigation of the unique surface (in real-world dimension) obtained by tremieying the concrete in different soil layers. An original and innovative procedure for concrete surface description was developed, which includes steps from the TLS measurement to the determination of the roughness parameters. The tested samples from anthropogenic soil, medium sand, and sandy gravel, map the real diaphragm wall surface. The surface roughness parameters in different soil layers were compared with the reference surface obtained by cast against steel formwork. The following parameters: *Sa*, *Sdr*, and *Vmc* are indicated as being the most useful in numerical description of the concrete surface type and in allowing the determination of the soil surface friction. The novelty of this study is the estimation of the parameter *δ* (friction angle between the retaining wall surface and the soil), which is, among others, a function of the wall surface roughness. The influence of the type of surface on earth pressure are generally recognized in laboratory tests. Based on the estimated in situ values of *δ*, the more reliable active and passive pressure coefficients *K_a_, K_p_* were calculated for the tested soil layers. The conducted study has a practical significance for designing of retaining construction and makes progress in determination of surface roughness required in Eurocode 7.

## 1. Introduction

One of the common applications of geodetic methods in relation to geotechnical structures is the measurement of displacements and deformations of soil or soil-structure systems. The widespread techniques and detailed descriptions of these studies are published, e.g., in [1,2,3]. Deformation analysis based on multitemporal Terrestrial Laser Scanning (TLS) surveys has been applied for many years in commercial and academic problem areas [4]. The close-range photogrammetry with image processing can be used to measure the ground deformation accurately [5,6]. Geodetic methods find direct application in the design process (observational methods) [7] as well as in structure monitoring and maintenance control [8,9,10,11]. Surveying results are frequently used to verify and calibrate analytical and numerical calculations of geotechnical structures [12]. Further branches of application of geodetic methods in geotechnics include surface testing to detect damages [13], determine thermal parameters [14] or surface roughness parameters [15,16]. The latter mentioned application is the subject of this article.

Typical reinforced concrete structures are formed using climbing shuttering, or a series of formworks that ensure controlled surface quality. In the case of reinforced concrete structures, the parameters of the surface roughness do not have a significant impact on the load-bearing capacity. They may be important for protection against corrosion or for aesthetic value. The control of surface parameters is easy to check. Concrete or reinforced concrete structures formed in the ground, such as piles, concrete columns, diaphragm walls, use the surrounding soil as a natural formwork. The parameters of surface roughness depend on the technology of forming, the type of soil, and its graining. Access to the underground surface is difficult or impossible. It is obvious that the surface roughness parameters affect the bearing capacity of the foundation piles [17]. The load transfer mechanism of shear forces between two layers: concrete and soil occurs thanks to the friction phenomenon, due to the existence of compression stresses at the interface and to the relative displacement between concrete and soil. As mentioned before, the access to the underground surface is difficult or impossible, therefore laboratory shear tests on the interface between coarse-grained soil and concrete conducted by, e.g., [18,19] are essential.

In the case of retaining structures, the influence of the type of surface on the effects of loading-earth pressure and resistance-passive earth pressure are generally recognized. In [20] the laboratory tests of the retaining wall model were carried out. Research has been conducted for active and passive retaining wall. The roughness of the wall was set in two types: rough and smooth. Results from image processing while the retaining wall moved due to earth pressure show that smooth surface condition generates greater displacement than the rough condition. In numerical analysis, when using virtual thickness factor and a *R_inter_* factor [21], these trends have been confirmed. The surface roughness characteristics in the above-mentioned research are only descriptive as well as in Eurocode 2 [22] and do not refer to the roughness parameters according to the standard [23]. Real retaining wall (as presented below in this work) is usually more complex than the concrete-soil samples prepared in the laboratory, due to the character of the surface of the diaphragm wall in variable geotechnical conditions.

**Figure 1:**
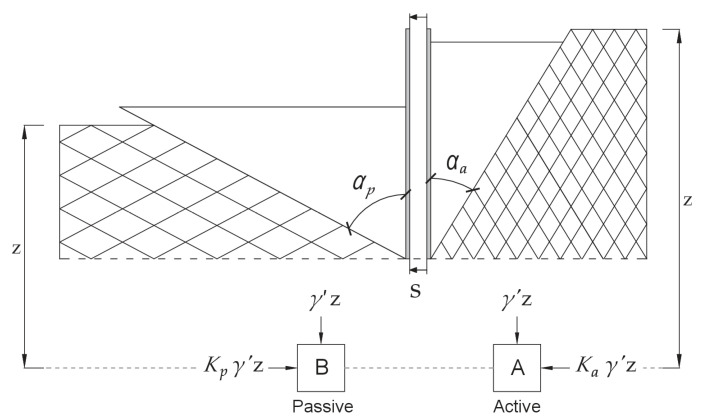
The active and passive sides of the wall along Rankine zones (courtesy of Sigurdur Mar Valsson [24]).

The correct estimate of lateral earth pressure is important for the design of earth retaining structures. The need to compare the various earth pressure equations with field measurements cannot be neglected. This comparison is not commonly done due to the difficulties associated with measuring the earth pressure itself. Understanding the behavior of soil-structure interaction and relationship between the roughness of the surface wall and the interface factors may contribute to design retaining walls safely and more efficiently. Basic concept of earth pressure is shown in Figure 1. The soil mass is bounded by a frictionless vertical retaining wall. A soil element at the depth of *z* is subjected to a vertical effective pressure and a horizontal effective pressure. There are no shear stresses on the vertical and horizontal planes of the soil element. Note that the horizontal earth pressure coefficient is valid for: vertical retaining wall, smooth wall in which the interface between the wall and soil is frictionless, the soil is homogeneous and isotropic. If there are only stresses in the soil caused by the self-weight of the soil (geostatic stresses), the major stresses occur in the vertical and horizontal directions. The stresses in horizontal plane are called stress in rest state. If the wall moves away from the soil mass and cannot maintain large stresses in the horizontal plane, the major stresses can be considered to be both vertical and horizontal. Stresses in horizontal plane are called active state.

Rankine zones and the stress fields drawn on either side of the wall in Figure 1 are called active and passive Rankine zones. A list of nomenclature for all the symbols can be found in the Appendix A. The failure wedge on the active zone will have inclination $\alpha_{a}=\left(45-\frac{\varphi^{\prime }}{2}\right)$ and failure wedge on the passive zone will have inclination $\alpha_{p}=\left(45+\frac{\varphi^{\prime }}{2}\right)$, where $\varphi^{\prime }$ is an angle of soil internal friction. The nondimensional quantity parameter describing soil pressure coefficient of pressure *K*, can be defined as ratio: horizontal pressure $\sigma_{h}^{\prime }$ to vertical pressure $\sigma_{z}^{\prime }$. This ratio is called the coefficient of Rankine’s active earth pressure and is given by

(1) $$K_{a}=\frac{\sigma_{h}^{\prime }}{\sigma_{z}^{\prime }}=tan^{2}\left(45-\frac{\varphi^{\prime }}{2}\right)$$

When the retaining wall moves into the soil towards the lower surcharge (left side), the soil wedge opposes. The effective principal stresses $\sigma_{h}^{\prime }$ will increase, and after reaching the ultimate state, the soil failure wedge is pushed upwards. The coefficient of Rankine’s passive earth pressure is given by:

(2) $$K_{p}=\frac{\sigma_{h}^{\prime }}{\sigma_{z}^{\prime }}=tan^{2}\left(45+\frac{\varphi^{\prime }}{2}\right)$$

As noted, the classical pressure Rankine theory assumes a smooth retaining wall surface. It is not plausible that all retaining structures should either be constructed as completely rough or completely smooth. To tackle the disadvantage of assuming a plane failure surface by Coulomb [25] and Rankine [26], Caquot et al. [27] developed a theory for earth pressure which was based on the logarithmic spiral theory.

In Annex C of the Eurocode 7 [28] the general equation for calculating the coefficient of earth pressure is given as:

(3) $$K=\frac{1\pm sin\varphi sin\left(2m_{w}\pm \varphi \right)}{1\mp sin\varphi sin\left(2m_{t}\pm \varphi \right)\;}\exp\pm \left(2vtan\varphi \right)$$

where:

(4) $$cos\left(2m_{t}\pm \varphi +\beta \right)=-\frac{sin\beta }{\pm sin\varphi }$$

(5) $$cos\left(2m_{w}\pm \varphi \pm \delta \right)=\frac{sin\delta }{sin\varphi }$$

(6) $$v=m_{t}+\beta -m_{w}-\theta$$

In work [24,29] is presented a single equation for both active and passive pressure.

(7) $$K_{p}=\frac{1+sin\varphi sin\left(2m_{w}+\varphi \right)}{1-sin\varphi sin\left(2m_{t}+\varphi \right)\;}\exp2\left(m_{t}+\beta -m_{w}-\theta \right)\left(tan\varphi \right)$$

where:

(8) $$m_{t}=0.5\left(arccos\left(\frac{-sin\beta }{sin\varphi }\right)-\varphi -\beta \right)$$

(9) $$m_{w}=0.5\left(arccos\left(\frac{sin\delta }{sin\varphi }\right)-\varphi -\delta \right)$$

(10) $$K_{a}=\frac{1-sin\varphi sin\left(2m_{w}-\varphi \right)}{1+sin\varphi sin\left(2m_{t}-\varphi \right)\;}\exp-2\left(m_{t}+\beta -m_{w}-\theta \right)(tan\varphi )$$

where:

(11) $$m_{t}=0.5\left(arccos\left(\frac{-sin\beta }{-sin\varphi }\right)+\varphi -\beta \right)$$

(12) $$m_{w}=0.5\left(arccos\left(\frac{sin\delta }{sin\varphi }\right)+\varphi +\delta \right)$$

where: φ = internal soil friction angle, β = is the slope angle of the ground behind the wall, δ *=* is the angle of shearing resistance between ground and wall, θ *=* the angle of inclination of the wall to the vertical.

**Figure 2:**
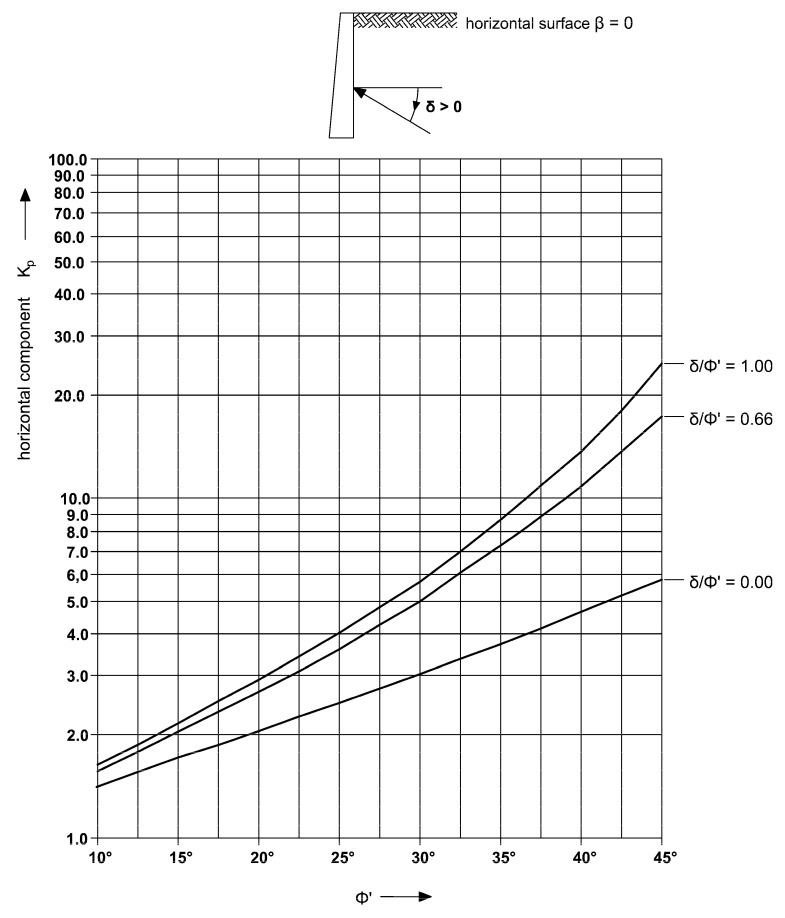
Coefficients *K_p_* of effective passive earth pressure (horizontal component): with horizontal retained surface (*β* = 0). Reprinted with permission from ref. [28]. 2008 Polish Committee for Standardization.

The parameter δ takes into account the roughness of the wall surface, but the standard [28] does not give details on how to take this value. Obtaining a rough surface is advantageous and leads to a reduction of active pressure on the retaining structure and increasing the passive resistance of the soil. The value of the parameter δ friction angle between the wall and the soil is a function of the surface roughness and the angle of internal friction of the soil. However, the standards [22,28] do not provide detailed recommendations on how to determine this parameter, they indicate a smooth, intermediate, and rough state, without linking them to the roughness surface parameters. Meanwhile, the roughness description is essential, especially when determining the passive earth resistance. For example, as shown in the diagram (Figure 2), for a typical value of the angle of soil internal friction *φ* = 30°, the value of passive earth pressure *K_p_* for the rough state is *K_p_* = 6.0 and for smooth state is *K_p_* = 3.0, which completely changes the design of the excavation protection in the soil and internal forces in it.

It is not possible to assemble the retaining structures as perfectly rough or perfectly smooth. The measurement of how much shear stress in the soil will be transferred to the structure, called the roughness ratio is defined as [30]:

(13) $$r=\frac{\tau }{\tau_{cr}}$$

where: *τ* = stands for the shear stress acting on the structure, and $\tau_{cr}$ = stands for the critical shear stress in the soil mass at failure. The roughness ratio will then take the value *r* = 0 when we have a completely smooth wall and the value when *r* = 1, the wall is completely rough. Parameter $\tau_{cr}$ = the critical shear stress in the soil mass, can be calculated according to the rules of soil mechanics, however, the parameter *τ* is a function of the internal friction angle of the soil and the stress normal to the surface, and the roughness of the surface. In typical mechanical systems, smooth surfaces of moving elements operate to reduce the friction force, resistance to motion, and wear, hence the expected values of roughness parameters that describe smooth surfaces. In the presented geotechnical problem, the surface roughness requirements are quite different. Obtaining a rough surface is favorable and leads to an increase in the load-bearing capacity of the retaining structure. As shown above in the code approach [28] and research [24], the precise definition and numerical roughness values remain unspecified. This is the motivation to determine the roughness parameters according to [23] for in situ concrete surfaces intuitively assumed as smooth, intermediately rough, and rough.

## 2. Materials and Methods

### 2.1. Terrestrial Laser Scanning

Terrestrial Laser Scanning (TLS) is a measurement technique that uses laser light to inquisition objects in three-dimensional space. Laser scanners can be classified according to various criteria, e.g., distance measurement, field-of-view, scanning resolution, measurement frequency, measurement range, and scanning speed [31]. In the literature, the most common categorization of scanners is based on the distance measurement method. The main types of scanning techniques mentioned in publications are pulse-based, phase-based, and triangulation-based [32,33,34].

In pulse-based scanners, the distance measurement is based on the parameter of the time of the laser flight from the emitter to the object and is reflected back to a sensor. The flight time of the laser is therefore measured for twice the distance. The distance d is obtained from the following Equation (14):

(14) $$d=\frac{c_{L}\cdot t}{2}$$

where: t = flight time of laser; $c_{L}$ = speed of light in the medium.

The phase-based method is based on a phase difference. The emitted laser light beam is amplitude modulated, which, reflected from the object’s surface, returns to the scanner sensor (receiver) with a defined time delay. Based on the dependence that the transition time of the signal is directly proportional to the phase difference of the wave, the distance d can be calculated as follows:

(15) $$d=\frac{c_{L}}{2}\cdot \frac{\varphi_{d}}{2\pi }\cdot T$$

where: $\varphi_{d}$ = phase difference between received and sent signal; T = period of the modulated signal.

The triangulation-based technique uses principles of the triangle to determine the location of measurement object. The measurement principle is described in detail by [35]. The laser emitter and the camera are set at a constant angle. Knowing the distance between the laser emitter and the camera (called the baseline), the angle of the laser emitter and the angle of the camera (which can be determined by looking at the position of the laser beam in the field of view of the camera), a triangle shape is obtained. This configuration enables the location of the measured object to be determined.

There are also other categorizations. According to [36], three electro-optical distance measurement techniques are detailed: time of flight, triangulation, and interferometry. As described in [31], in the time-of-flight method there can be differentiate:

- the direct time of flight (pulsed time of flight the equivalent of mentioned pulse-based method),
- the indirect time of flight (amplitude-modulated continuous wave, frequency-modulated continuous wave, and polarization modulation).

Interferometry is a distance determination method based on interfering electromagnetic waves. The essence of measuring interferometric scanners is presented, among others, by [35]. The laser beam is divided into two parts: half of the laser beam is reflected by the beam splitter and the other half is transmitted. When both parts are connected together, interference pattern are created. The analysis of the interference patterns allows the determination of the distance.

### 2.2. Study Site

The study site was located on a construction site of an office building in Wrocław, Poland. The city is situated on The Silesian Lowland which is the southernmost part of the Middle-Polish Lowlands. There is a vast plain with little diversity of relief. It spreads from the southeast to the northwest, along the glacial valleys of the Oder River, which is filled with alluvial sediments of Pleistocene and Holocene, mostly sand and gravel [37]. For decades, the area of the city has undergone intensive processes of urbanization, a constant influx of people, the development of processing industry, and the damage from military conflicts, and reconstruction afterwards. These activities became the reason for changes in the natural environment, especially in the subsoil. The anthropogenic changes take place on the surface of the terrain where they have impact on the civil structures.

**Figure 3:**
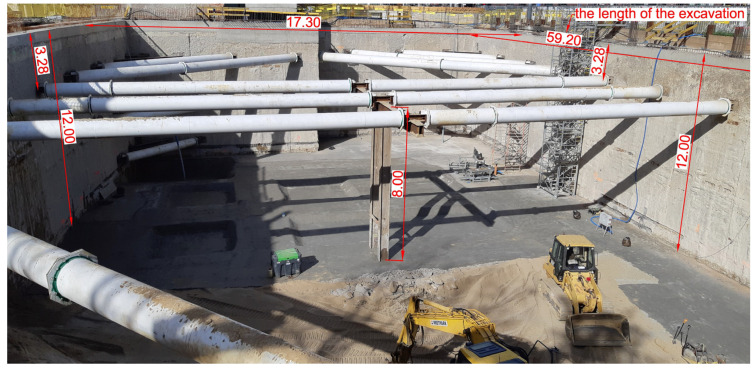
Protection of deep excavation by means of diaphragm wall and struts system.

Diaphragm walls are mainly designed and installed at recent excavation works in urban area because of some benefits such as the high stiffness of walls and the reducing of construction period [38]. Figure 3 shows the considered excavation work site using diaphragm walls supported by struts system. The diaphragm wall with thickness of 80 cm was installed at the excavated depth ranged to 12 m. The bottom of the wall was sunk 5.5 m below the excavation depth in cohesive soils. The planned underground part of the building will include three levels of a car park.

**Figure 4:**
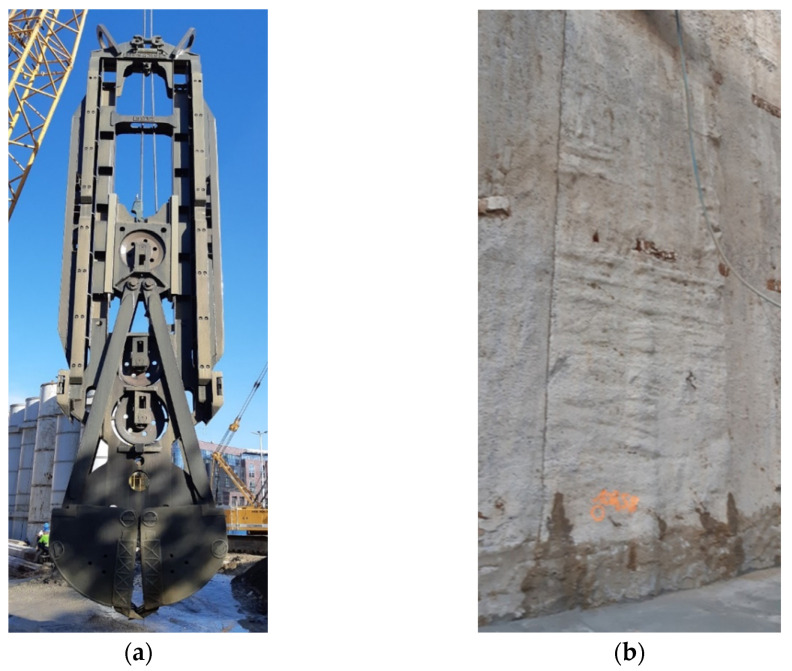
Photos from the construction site: (**a**) Cutter excavation machine; (**b**) The influence of technology on the concrete surface.

Before starting the excavation, a bentonite slurry plant must be installed for mixing and providing bentonite to the excavated panels through pipes. The guide walls that will prevent the soil from collapsing at the top of the designed wall, and will facilitate the installation of bentonite pipes that will deliver bentonite to the excavated panel, should be done initially. Special device clamshells, also known as grabs/buckets/cutters, are rectangular shaped and used to excavate vertical panels (Figure 4a). Loose or medium compacted sand and gravels can be excavated by using grab while excavation of hard soils strata can be processed by using cutter. The digging device can be cable or hydraulically driven. In the second phase of the technology, a vertical panel with a finite length and designed depth is excavated under the hydrostatic support from inside the bentonite slurry. During excavation, the bentonite must be fed to the panel simultaneously, while the excavated soil with bentonite is sent to the bentonite slurry plant for recycling [39]. End stops are used to control concrete placement so that secondly adjacent panels are not excavating monolithic concrete. Prior to tremieying the concrete, and while the panel is excavated the bentonite slurry contaminated by soil particles is simultaneously pumped out and cleaned. The bentonite slurry within acceptable parameters (density, sand content, viscosity, and PH) is fed to the panel. Cleaning and desanding slurry decrease its density so that tremie concrete doesn’t mix with slurry or native soil. Slurry is circulated at regular intervals throughout the construction period through the regeneration plant. Reinforcing cage is inserted into finished excavated panel, while the bentonite slurry still has a stabilizing effect. The final step assembling of diaphragm wall is tremie concreting, the technological process that involves replacing supporting slurry with concrete. With the vertical pipes called tremies, concreting starts from the bottom and the tremies are lifted steadily increasing as the top level of the wall rises.

Each of the presented technology stages of wall formation influences the final shape, roughness, and parameters of the wall surface roughness. The visible rough, heterogeneous surface of the wall results from the graining of the concrete and also the graining of the native soil layers in which wall is made. The roughness resulting from the graining will be visible in detailed tests with a laser scanner, while the vertical and horizontal waviness visible in the photo is a result of the technology making the wall. The vertical waviness reflects the vertical elements of the bucket frame structure (Figure 4a), and the numerous horizontal wavy lines (Figure 4b) are the traces of the bucket’s successive strokes during free fall into the native soil, they are the ordinates of the bucket’s successive hollows. Slurry cleaning and desanding removes finer fractions from the native soil in the soil-concrete interface. The influence of the latter waviness on the earth pressure coefficients should be considered in laser scanning data processing.

### 2.3. Geotechnical Conditions

**Figure 5:**
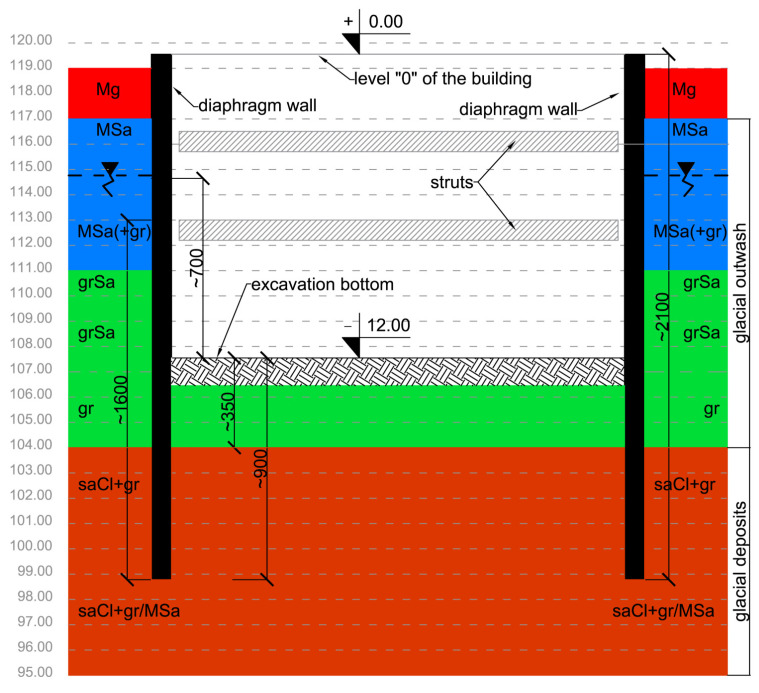
Diaphragm walls supported by struts the cross-section of the excavation and geotechnical conditions.

A geotechnical cross-section of the excavation site is presented in Figure 5, where Mg is anthropogenic soil, MSa is medium sand, gr is gravel, grSa is sandy gravel, and saCl is sandy clay. The ground is composed of anthropogenic soils and sedimentary soils. After the excavation has been made, slurry wall surfaces in non-cohesive, saturated soil are accessible. Geotechnical conditions are favorable, the slurry wall sinking in low permeable cohesive soils prevents the inflow of groundwater into the excavation.

### 2.4. Geodetic Measurements

**Figure A1:**
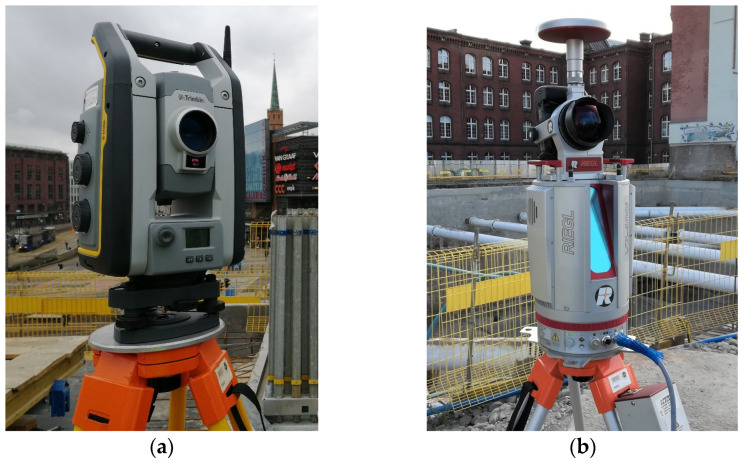
Geodetic instruments used in the course of the measurements: (**a**) Trimble S7 robotic total station; (**b**) Riegl VZ-400i laser scanner.

**Figure A2:**
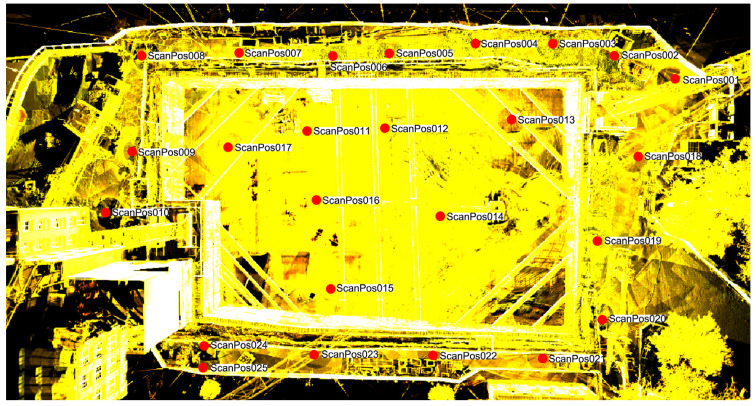
Distribution of scanner stations on the construction site.

For the purposes of a wider research project, a network of geodetic points was established on the construction site and its surroundings. The network consisted of nine reference points, five instrument stations, and 49 tie points. Measurements were made with a Trimble S7 robotic total station (Figure A1a—Appendix B). Each point was measured in at least two series, and in each series the measurements were made in two positions of the telescope (face 1 and 2). The angular-linear observations were adjusted with the least square method in the adopted local reference frame. After adjustment, the mean square error of point position was equal 1.45 mm, and do not exceed 2.1 mm for the worst determined point. The mean square error of height was equal 1.23 mm, and do not exceed 1.8 mm for the worst determined point. Laser scanning of the entire construction site was performed with a Riegl VZ-400i pulse scanner (Figure A1b—Appendix B) from 25 positions. Seven scanner positions were located at the bottom of the excavation (marked as: ScanPos11—ScanPos17), and the remaining positions were at the ground level around the excavation (Figure A2—Appendix B). Panoramic scan with a resolution of 20 mdeg, scan of visible tie points signaled by reflective sheet targets, and series of wide-angle photos from the integrated camera were performed at each scanner position.

**Figure 6:**
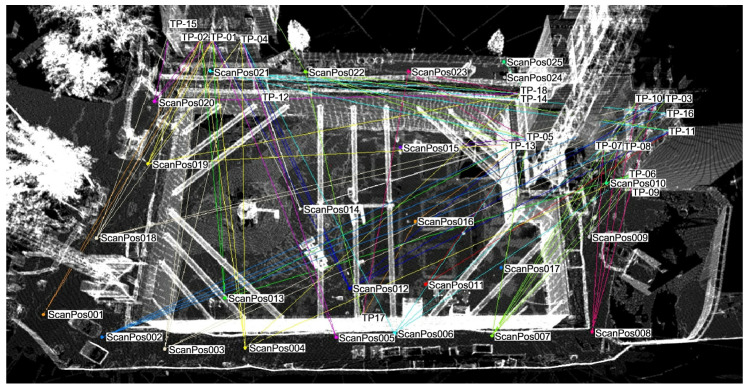
Registration and mutual alignment of point clouds with visible directions to tie points.

**Figure A3:**
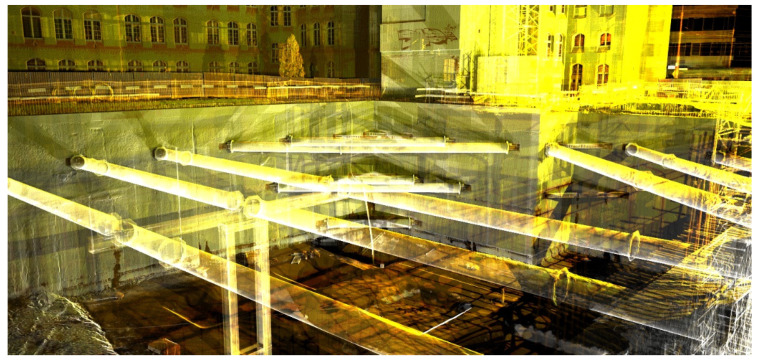
The view of point cloud after processing.

The initial processing of data from the laser scanner consisted in manually checking the point cloud from each station, and removing unnecessarily scanned people and incorrectly detected tie points. Then the point clouds were filtered with the use of reflectance and deviation parameters, which allowed to remove most of the measurement noise and false reflections. Subsequently, the initial registration of point clouds from individual scanner positions was performed based on data from the GNSS receiver and Inertial Measurement Unit (IMU). The final combination of point clouds was carried out by mutual alignment of the common surfaces also considering the tie points (Multi-Station Adjustment in RiSCAN PRO software [40] Figure 6). Afterwards, the merged point cloud from all scanner positions was fitted to the target local coordinate system based on the known coordinates of the tie points. The mean error of georeferencing process was equal 2.2 mm. A fragment of the point cloud after processing is shown in (Figure A3—Appendix B).

**Figure 7:**
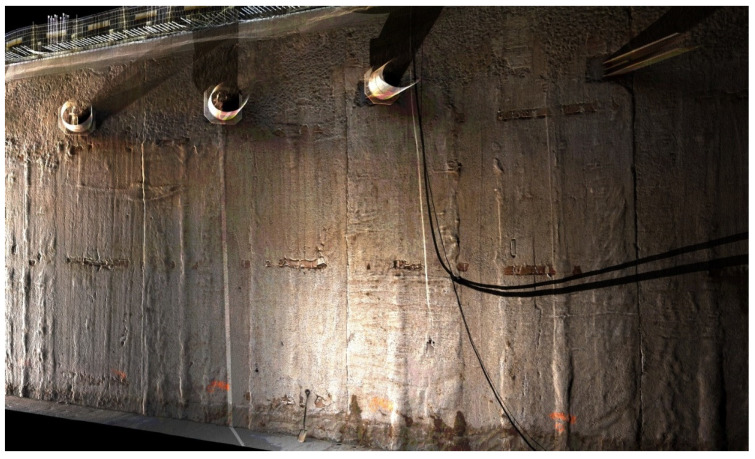
View of the point cloud (in the RGB color space) of northern diaphragm wall.

The northern diaphragm wall was selected for further research due to its greater availability. The obtained point cloud was very dense, as shown in Figure 7.

**Figure 8:**
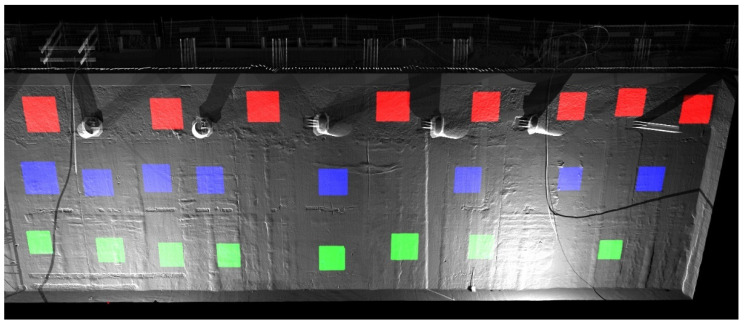
Location of measurement samples on the diaphragm wall in individual geotechnical layers: red color samples from Mg (anthropogenic soil), blue color samples from MSa (medium sand), green color samples from grSa (sandy gravel).

For each geotechnical layer, eight samples of 1 × 1 m dimensions were cut out from the point cloud representing the surface of the excavated diaphragm wall in convenient places (Figure 8). Samples marked in red come from anthropogenic soil (Mg), samples marked in blue from medium sand (MSa), and samples marked in green from sandy gravel (grSa) layer. The sample designation consisted of a symbol describing the geotechnical layer and the sequence number of the sample within the layer, numbered from right to left (Figure 8).

**Figure 9:**
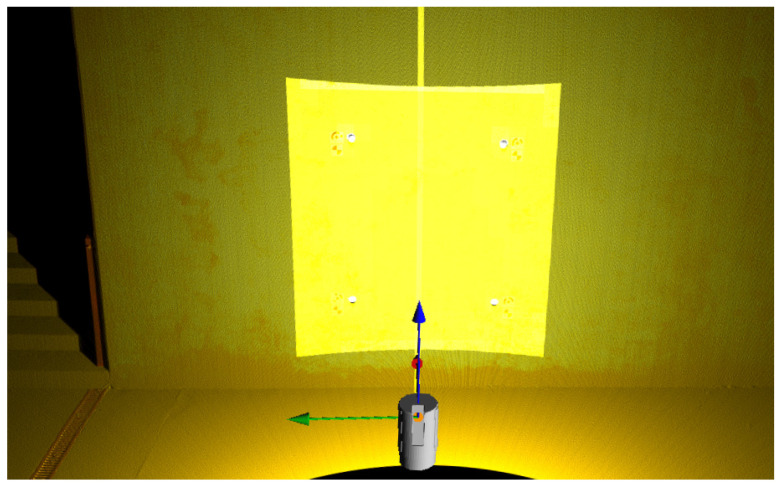
View of point cloud of reference wall (concrete surface made in the formwork).

To compare the surface of the diaphragm wall with a smooth concrete surface that could be treated as a reference surface, a measurement of a fragment of a concrete wall made in the formwork was carried out (Figure 9). The same measuring instruments and an analogous method of processing the point cloud were used. For further processing, two samples of 1 × 1 m dimensions were cut from the point cloud, hereinafter referred to as “reference surface” and numbered as RS1, RS2.

### 2.5. Procedure for Determination of Surface Parameters

In the literature, the researchers were particularly interested in the parameters of pile surface roughness. In paper [41], a series of pullout tests were conducted on a model pile in a soil with different initial water contents and different pile surface roughness (smooth or rough) to study their influences on pile skin friction. Smooth surface was considered as mild steel and rough as screwed surface. The normalized surface roughness (*R_n_*) of the interface is based on the roughness profile proposed in work [42] in Equation (16):

(16) $$R_{n}=\frac{R_{max}}{d_{50}}$$

where: $R_{max}$ = maximum peak to valley height, in code [21] parameter is described as *S_z_*; *d*_50_ = grain size (diameter) corresponding to 50% finer. For steel surface $R_{max}$ was measured as 0.0025 mm.

The $R_{max}$ parameter is one-dimensional and can be used for slim piles, however, for the diaphragm wall, the 3D parameters, the graining of the concrete aggregate and the native soil should be considered. The analysis of the surface morphology in the context of scientific and engineering applications is more and more often carried out in 3D space [19,43,44,45]. Using specialized software, it is possible to generate isometric 3D views of the tested surface and obtain roughness parameters [46]. The terms, definitions and parameters of the surface geometry are specified in detail in the ISO 25178 series of standards. Depending on their functionality, roughness parameters can be divided into several groups [44,47]: height parameters, spatial parameters, hybrid parameters, functional parameters, feature parameters, other 3D parameter. Due to the large number of parameters, the “parameter rash” phenomenon is observable, causing problems with the selection of appropriate 3D morphological parameters for surface description, including the concrete surface. Attempts to classify useful parameters for assessing the morphology of the concrete surface were made in the works, among others, in [48,49]. The selected parameters are presented in Table 1.

In general, for evaluation of area *A* the spatial distribution of ordinates Z is analyzed at a specific sampling density of this area (ordinate measurement using various techniques). The maximum height Sz is a vertical distance (along *Z* axis) between the maximum peak height and the maximum valley depth within *A* area. Parameter Sa is an arithmetic mean of absolute values of *Z* (height) within evaluation area. Parameter Sq shows the typical magnitude of *Z* (height), regardless of the sign of heights. The skewness Ssk evaluates deviations in the height distribution. When distribution is symmetrical then Ssk=0. Values above zero indicate the predominance of rounded peaks on the sample surface. Values below zero indicate the predominance of sharp peaks. The kurtosis Sku evaluates sharpness in height distribution. For normal distribution Sku=3. Values above 3 indicate sharp distribution (the surface resembles a zigzag). Values below 3 indicate even distribution (the surface resembles a sine wave). Parameter Sdr determines the increase of the surface area in relation to the area of its projection on the plane. Functional volume parameters describe the contribution of core, peaks, and dales in material surface on the basis of the material ratio curve with 10% and 80% as typical threshold [51].

**Figure 10:**
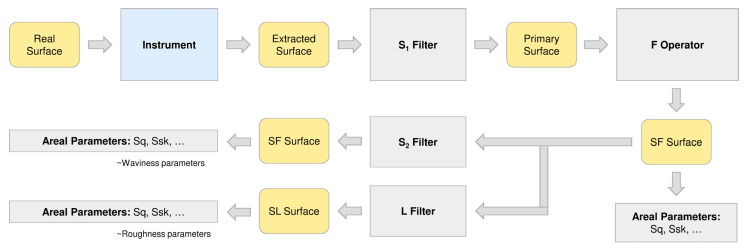
Typical procedure for areal parameters determination (courtesy of François Blateyron, Digital Surf [52]).

A typical procedure for areal parameters determination, according to standard [23], was described in [52] (Figure 10). The actual surface of the sample is mapped using a specific measuring instrument, with limitations resulting from this instrument’s specificity. The result is referred to as an extracted surface. Then, the S_1_ filter could be applied for removing measurement noise. The next step is to level the sample, which is denoted as F operator, and the resulting surface is named as SF surface. Then it is possible to first determine the searched areal parameters. Any surface can be composed of different geometrical structures with different scales [53]. A surface with a fine structure (roughness) has a small scale. The waviness, as a periodic component, can be small-scale and large-scale. The essence of subsequent filtration is to extract small-scale and large-scale fragments, respectively, from the surface measurement data. The S_2_ filter is a low-pass filter and is responsible for the elimination of short-wave surface deviation. The L filter, on the other hand, is a high-pass filter that removes long-term surface deviation. After separating waviness from roughness, the second set of areal parameters should be determined. This procedure is typical primarily for surface testing in mechanical applications where friction must be eliminated.

**Figure 11:**
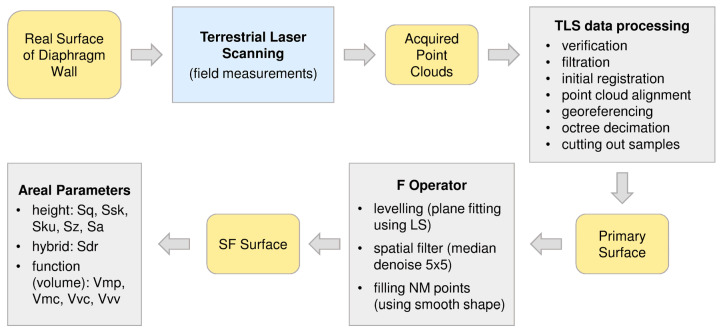
The procedure for the assessment of roughness of the diaphragm wall proposed by the authors.

For the assessment of roughness of the diaphragm wall (on the basis of the laser scanning measurement) for the needs of earth pressure estimation, the original procedure was proposed by the authors (Figure 11).

The first stage is to plan the field measurements. In the case of using laser scanners which do not require leveling, it is worth setting up a geodetic network of tie points, using a total station, in order to accurately determine the vertical axis of the coordinate system. Accuracy of inertial sensors of the scanner may be insufficient. When it is necessary to geo-reference the point cloud, establishing of tie points is almost obligatory.

The second stage is TLS data processing. Some of the processing steps are typical but must be adapted to the type of scanner and the specifics of the measured object. During filtering, the authors eliminated points with reflectance below −16 dB, and signal deviation above 15 in RiSCAN PRO software [40]. Some steps are described in detail in the Section 2.4. The original proposition of the authors is to use of the octree method for samples decimation. The combined point cloud has an irregular point distribution in the sample area. Additionally, the measurement noise of the scanner, caused by the inaccuracy of the distance measurement with the use of a reflector-less rangefinder, causes a certain “thickness” of the point cloud surface representing the concrete surface. Depending on the accuracy of the scanner, this “thickness” varies within a few millimeters. The authors propose to perform an octree decimation with cell dimensions of 5 × 5 mm “along” the wall, and 50 cm “perpendicular” to the wall. In each octree cell, one real measured point should be left, closest to the average position of all measured points which are in the cell. The 5 mm cell side corresponds to the approximate size of the laser spot falling on the object during the measurement. 50 cm high of the cell considers any unevenness of the diaphragm wall. The use of octree decimation ensures regular distribution of points “along” the wall and eliminates “thickness” of the surface maintaining the characteristic topography of the sample surface. After decimation, the samples were trimmed to the target size (in CloudCompare software [54]), which was a square with a side of 1 m (treated as the primary surfaces).

The next steps were performed in Mountain Maps software [55]. At the beginning, each sample was leveled using least square planes (LSP) through subtraction. Then the samples were spatially filtered to minimize the measuring noise. A 5 × 5 mm median filter was used to denoise. In the next step, all not measured (NM) points were filled with a smooth shape calculated according to the neighbors. Then, the set of chosen areal parameters were calculated.

To assess the roughness of concrete interacting with the ground in terms of the estimation of earth pressure the greatest unevenness of the surface is of the greatest importance. Therefore, according to the authors, the further steps shown in Figure 10 should not be followed (S_2_ filter and L filter). In the case of a diaphragm wall, separating the waviness from the roughness would lead to unnecessary splitting of the overall effect into parts.

## 3. Results

**Figure 12:**
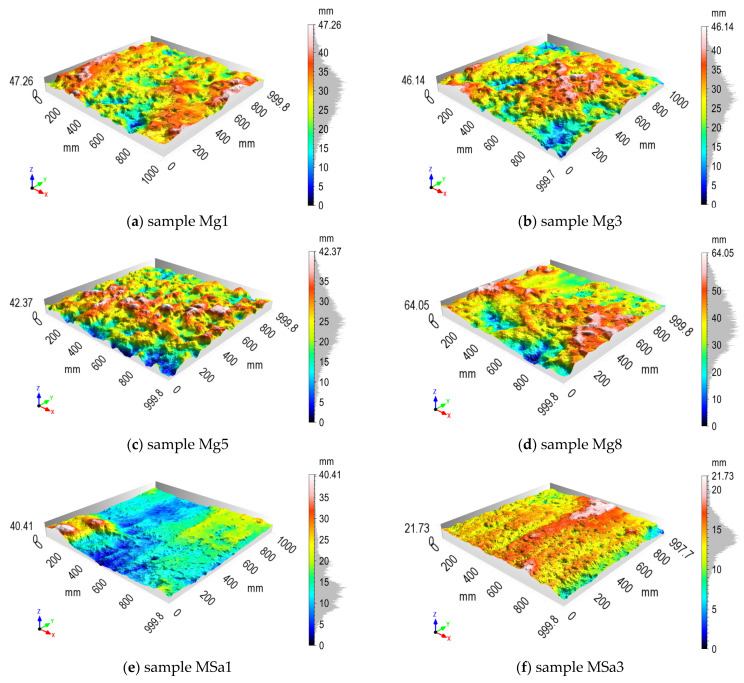

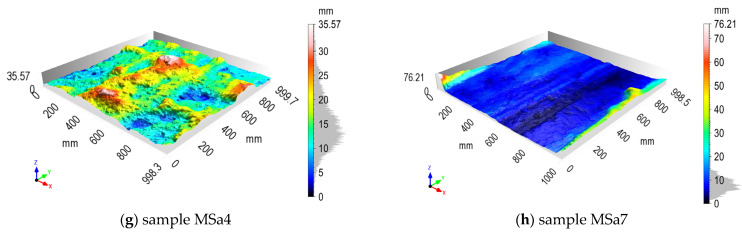
Results of diaphragm wall surface processing for chosen samples: (**a**–**d**) from anthropogenic soil; (**e**–**h**) from medium sand.

**Figure 13:**
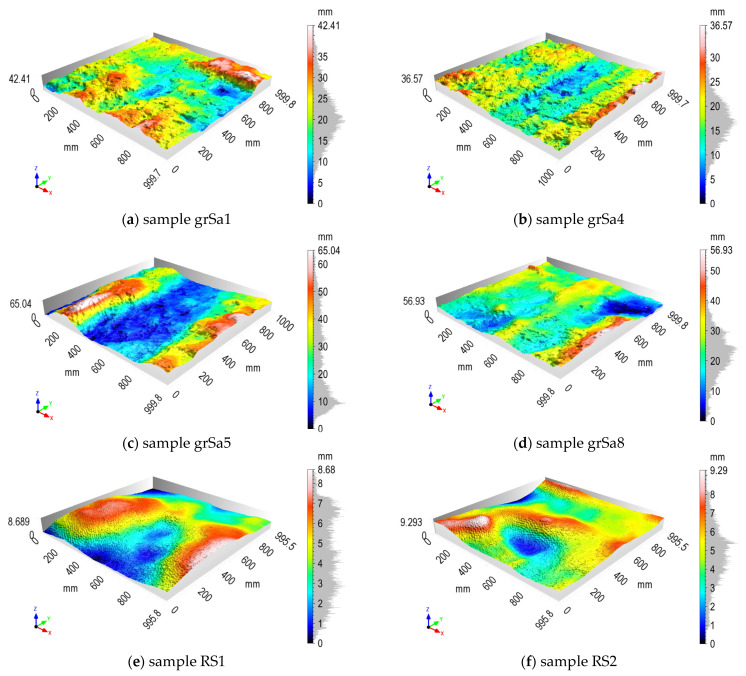
Results of surface processing for chosen samples: (**a**–**d**) diaphragm wall from sandy gravel; (**e**,**f**) reference surface.

As was presented in Figure 8, the eight samples were processed for each geotechnical layer. 3D models of chosen samples for anthropogenic soil and for medium sand are presented in Figure 12, as well as for sandy gravel and for reference surface in Figure 13, respectively. The samples from anthropogenic soil are characterized by a large number of fine but fairly evenly distributed irregularities. The samples from medium sand and sandy gravel are very diverse, even in the same layer. The reference wall samples are much flatter, which is not surprising.

**Figure 14:**
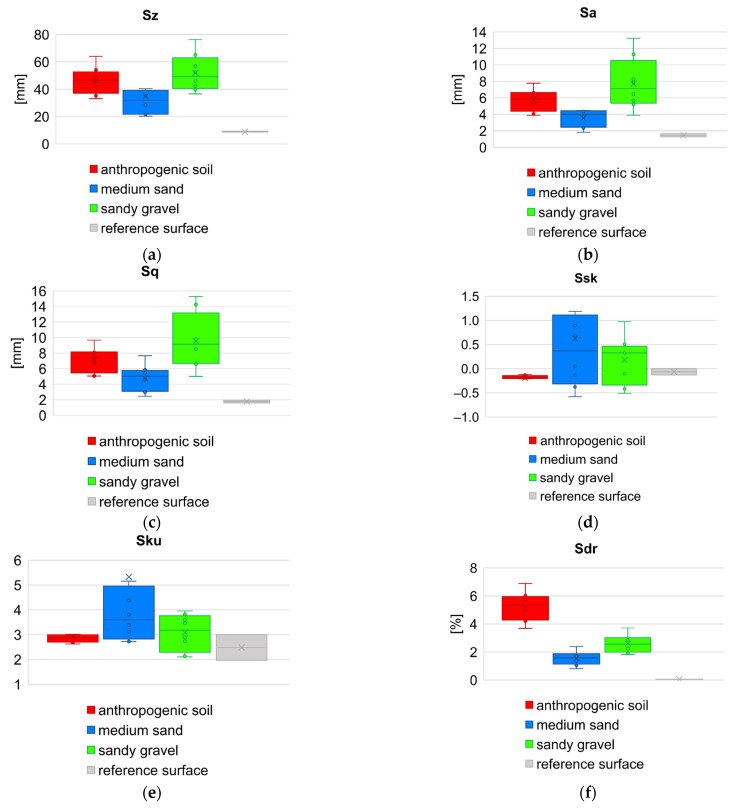
Box plot comparison of height and hybrid parameters of the concrete surface: (**a**) maximum height; (**b**) arithmetical mean height; (**c**) root mean square height; (**d**) skewness; (**e**) kurtosis; (**f**) developed interfacial area ratio.

**Figure 15:**
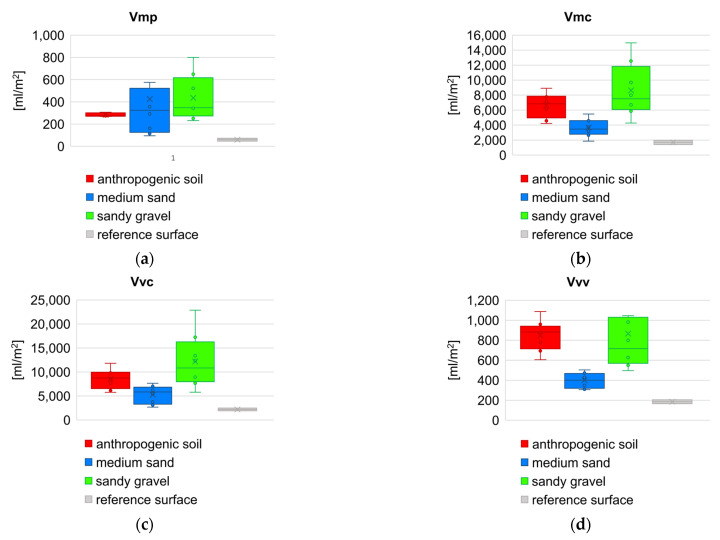
Box plot comparison of functional volume parameters: (**a**) peak material volume; (**b**) core material volume; (**c**) core void volume; (**d**) dale void volume.

**Figure 16:**
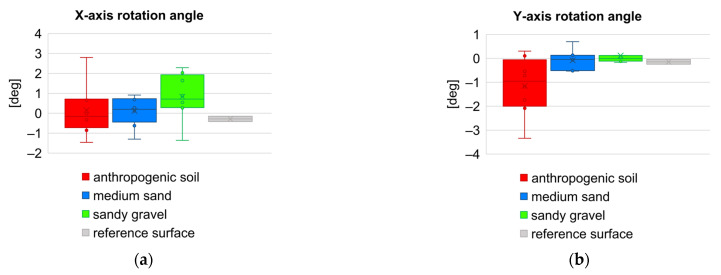
Box plot comparison of inclination angles of the wall surface: (**a**) deviation from verticality; (**b**) deviations from straightness (along the diaphragm wall).

In order to compare the values of the roughness parameters between respective geotechnical layers, as well as to show the variability of these parameters within a given layer (on the basis of eight samples for each layer), the set of box plots were prepared. Comparison of results for height and hybrid parameters is juxtaposed in Figure 14, and for functional volume parameters in Figure 15, respectively. Figure 16 presents box plot comparison of inclination angles of the wall surface in two directions: deviation from verticality, and deviations from straightness (along the diaphragm wall).

## 4. Discussion

The influence of the surface type of a retaining structure on the loading (active earth pressure) and soil resistance (passive earth pressure) are generally recognized in laboratory tests. In [20], the roughness of the retaining wall was set in two types: rough and smooth. It does not consider more advanced variety of subsoil, which modify the concrete surface roughness. The currently conducted research is aimed at determining displacements and deformations as well as the detection of the occurrence of limit states. The investigations presented in [9,56] allow for the check of global behavior of retaining structures in the field of structural health monitoring. Otherwise, the research in this paper uses geodetic methods (TLS) to describe in situ surface roughness parameters and further use them to more precise calculations of geotechnical loads earth pressure on the retaining structure.

The research object (diaphragm wall) is made of concrete with a controlled grain distribution and therefore it can be assumed that the presented variability of the surface parameters results from the execution of a slurry in soils with different grain sizes. Soil graining (particle size composition) is determined by the percentage content of individual fractions in relation to the weight of the entire tested soil sample [57]. The percentage of each fraction is necessary to determine the type of soil. Soil graining is characterized by the index U:

(17) $$U=\frac{d_{60}}{d_{10}}$$

where: *d*_10_, *d*_60_ = denote the particle diameters, which together with the smaller ones constitute 10, 60% of the mass of the sample, respectively.

Sandy gravel is well graded with parameter value U > 15; gravel and medium sand are uniform graded with U > 6; gravel is poorly graded with U > 4. Sandy gravel contains coarse, sandy, and fine (silty) grain; medium sand contains sandy grain with some gravel.

As shown in the geotechnical profile (Figure 5), there are soils that consist of various grains as well as non-cohesive and fine-grained (cohesive) soils. The dimensionless roughness can be also estimated from the Equation (16). Grain size distribution be used here to explain the difference in roughness parameters obtained by diaphragm wall surface. The parameter value *S_z_* is biggest for sandy gravel, while according to grain size distributions for sandy gravel and medium sand, the diameter *D*_50_ is comparable for both layers, hence the dimensionless roughness *R_n_* is greater for sandy gravel. In the case of the *S_z_* parameter, its values are determined by the extreme values from the height distribution of the sample (the tails of height distribution). When analyzing larger areas (samples about 1 m^2^), the *S_a_* and *S_q_* parameters are much more reliable than the *S_z_* parameter, because *S_a_* represent the arithmetic mean of the absolute sample’s height within the evaluation area, and *S_q_* represents the root mean square of sample’s height within the evaluation area, respectively. Additionally, these parameters are not significantly influenced by scratches, contamination, and measurement noise [51]. Considering Figure 14b,c, the overall distribution of parameter values is similar to grain size distribution of soil. As expected, the values of the *S_a_* and *S_q_* parameters for the reference surface are very small and are at the level of the scanner measurement errors as well as the inaccuracy of the formwork flatness. Parameter *S_sk_* is used for deviation evaluation in the height distribution. For the reference surface the height distribution is almost symmetric against the mean line. For anthropogenic soil the height distribution is slightly deviated to the upper side, but for medium sand and sandy gravel the height distribution is slightly deviated to the lower side. Approximately, all geotechnical layers and reference surface have normal height distribution (*S_ku_* ≈ 3). Only medium sand has a slight tendency to sharp height distribution.

The *S_dr_* parameter of a completely level surface is 0. This is the case of reference concrete surface. When a surface has any slope, its *S_dr_* value becomes larger. The parameter value *S_dr_* is higher for sandy gravel then for medium sand. Then there is a “notched joint” connection between concrete and soil. A similar relationship exists for the *V_mp_* parameter, which indicates greater roughness for sandy gravel. The estimation *δ* angle, as a function of the soil friction angle *φ* only, as it is shown in the Figure 2, is therefore not inaccurate. In particular, for soil layers with the same friction angle value, the same roughness is assumed, and the grain size effect is neglected. The variability of the sample inclination angles in both directions (Figure 16a,b) results from the random distribution of the samples on the surface of the diaphragm wall and the traces of the cutter work. The sample size (1 square meter) is much smaller than the width of the cutter, which excavates a 3 m wide panel during the work cycle (Figure 7 and Figure 8).

Anthropogenic soils that occur in urban areas as layers of large thickness, for which the angle of internal friction is usually not determined in laboratory tests, and they are treated as non-bearing or weak soil. The research conducted in [58] an adjacent location indicates that construction debris and dumps in urbanized areas, which were built-up in the past, can be treated as non-cohesive soils. In order to indicate the influence of the earth pressure, the values *K_a_, K_p_* were estimated in Table 2 for smooth surfaces and in Table 3 for rough surfaces according to [28] for horizontal retained surface (*β* = 0). Internal soil friction angle *φ* of soil layers are adopted on the basis of geotechnical investigation.

The values of passive pressure, which are a function of the coefficients *K_p_*, are sensitive to the change of numerical parameters, therefore, considering the surface roughness of the retaining here significant.

The *fib* Model Code 2010 [59,60] present an improvement related with the explicit contribution of each load transfer mechanism cohesion, friction and dovel action in the design expression and with the characterization of the surface roughness. This code adopts a roughness parameter the average roughness (*R_a_*) to describe the surface roughness of the concrete substrate. Depending on the treatment method and the calculated average roughness parameter (*R_a_*) the roughness is classified according to four categories: (1) very smooth, surface obtained by cast against steel formwork, *R_a_* is not measurable; (2) smooth untreated surface, obtained by cast against wooden formwork, *R_a_* < 1.5 mm; (3) rough, surface obtained by sand blasting or similar treatment *R_a_* ≥ 1.5 mm; (4) very rough surface obtained by high pressure water blasting or similar treatment or intended surfaces *R_a_* ≥ 3.0 mm. These standards do not consider the surface of concrete formed by cast against soil layer. In the conducted 3D tests, the parameter *S_a_* is the equivalent of mentioned in the code *R_a_* parameter. Based on the received parameter S*_a_*, *S_z_*, *S_dr_*, *V_mp_*, *V_vc_* values, the diaphragm wall surfaces may be described as rough/very rough and reference surface as smooth/very smooth. The task is to determine the value of the angle considering the roughness value of the concrete surface. If the coefficient of friction is adopted from the standard [22], then *μ* = 0.8 for rough surface. Maximum shear stress in the soil at the contact surface is defined as:

(18) $$\tau_{cr}=\gamma \cdot z\cdot K\cdot tg\varphi$$

where: K = is coefficient of effective horizontal earth pressure; γ = the total weight density of retained ground; z *=* distance down the face of the wall.

Shear stress in the soil at rough contact concrete surface is given by:

(19) τ=μ ·γ·z ·K· tgφ

Then angle *δ* is calculated from the relationship:

(20) $$tg\delta =\frac{\tau }{\sigma_{h}}=\frac{\mu \cdot \gamma \cdot z\cdot K\cdot tg\varphi }{\gamma \cdot z\cdot K\;}=\mu \cdot tg\varphi$$

(21) δ=arctg(μ·tgφ)

The values of angle δ for subsequent layers and calculated on this basis earth pressure coefficients *K* with horizontal retained surface (*β* = 0) are listed in Table 4.

The value of the angle *δ* on the basis of the above performed calculations gives an advantageous change the pressure coefficients in the range of 5–10%, in particular passive earth pressure.

## 5. Conclusions

The article presents the application of laser scanning for concrete surface morphology assessment. Two types of concrete surfaces differing in the manufacture method were examined: very smooth concrete surface obtained by cast against steel formwork, as well as rough/very rough surface obtained by tremieying the concrete in different soil layers. Acquiring these data is difficult due to the formation of diaphragm wall in the ground and their subsequent work as an underground structure.

The roughness characteristics in the Eurocode 2 [22] are only descriptive and do not refer to the roughness parameters according to the standard [23]. The tested samples describe/map the real diaphragm wall surface and allow to describe numerically the type of concrete surfaces. The authors proposed an innovative and comprehensive procedure for acquiring data on the roughness of the surface of a concrete diaphragm wall on a construction site (in real-world dimensions). The proposed procedure includes steps from planning the measurement with a laser scanner at the construction site to determining the roughness parameters of the concrete surface and can be applied to other concrete geotechnical structures.

The application of proposed procedure allows for:

- determination of the values of significant parameters of surface roughness;
- separation of geotechnical layers with various roughness;
- estimation of the parameter *δ* (friction angle between the retaining wall surface and the soil), which is a function of the wall surface roughness and the angle of internal friction of the soil;
- more reliable calculation of earth pressure in separate geotechnical layers;
- obtainment of roughness parameters with practical significance for earth active and passive pressure calculation.

This study can be enhanced in further research by increasing the number of surface samples and including cohesive soils to predict earth pressure values more reliable based on the geodetic measurement techniques.

## Figures and Tables

**Table 1:** Examples of surface parameters and their definitions according to [23,50].

Name of Parameter	Definition
Height parameters	
Root-mean-square height	$Sq=\sqrt{\frac{1}{A}\iint_{A}{\left(Z\left(x,y\right)\right)}^{2}dxdy}$
Skewness	$Ssk=\frac{1}{Sq^{3}}\left[\frac{1}{A}\iint_{A}{\left(Z\left(x,y\right)\right)}^{3}dxdy\right]$
Kurtosis	$Sku=1/Sq^{4}/A\;\iint_{A}Z{\left(x,y\right)}^{2}dxdy$
Maximum height	$Sz=sup\left\{Z\left(x_{i},y_{i}\right)\right\}+\left|inf\left\{Z\left(x_{i},y_{i}\right)\right\}\right|$
Arithmetic mean height	$Sa=1/A\;\iint_{A}\left|Z\left(x,y\right)\right|dxdy$
Hybrid parameters	
Developed interfacial area ratio	$Sdr=\frac{1}{A}\left[\iint_{A}\left(\sqrt{\left[1+{\left(\frac{\partial z\left(x,y\right)}{\partial x}\right)}^{2}+{\left(\frac{\partial z\left(x,y\right)}{\partial y}\right)}^{2}\right]-1}\right)dxdy\right]$
Functional Volume Parameters	
Peak material volume	Vmp=Vm(p)
Core material volume	Vmc=Vm(q)−Vm(p)
Core void volume	Vvc=Vv(p)−Vv(q)
Dale void volume	Vvv=Vv(q)

**Table 2:** Earth pressure coefficient *K_a_, K_p,_* for smooth surface according to [28].

Surface	*φ*	*δ*	*K_a_ δ* = 0.00	*K_p_ δ* = 0.00
	[°]	[°]	[–]	[–]
concrete/Mg	29	0	0.36	3.0
concrete/MSa	33	0	0.33	3.2
concrete/grSa	38	0	0.24	3.5

**Table 3:** Earth pressure coefficient *K_a_, K_p,_* for rough surface according to [28].

Surface	*φ*	*δ*	*φ / δ*	*K_a_ δ/φ =* 0.66	*K_p_ δ/φ* = 0.66
	[°]	[°]	[–]	[–]	[–]
concrete/Mg	29	19.1	0.66	0.30	4.3
concrete/MSa	33	21.7	0.66	0.26	5.6
concrete/grSa	38	25.0	0.66	0.20	8.1

**Table 4:** Pressure coefficient values *K_a_, K_p_* for tested surfaces according to Equations (7) and (10).

Surface	Surface Parameter (Average)					Surface Type	*δ*	*K_a_*	*K_p_*
	*S_a_*	*S_z_*	*S_dr_*	*V_mp_*	*V_vc_*				
	[mm]	[mm]	[%]	[mL/m²]	[mL/m²]		[°]	[-]	[-]
reference concrete	1.5	9.0	0.1	60	2198	smooth/very smooth	0	-	-
concrete/Mg	5.8	46.4	5.2	284	8587	very rough	23	0.29	4.5
concrete/MSa	3.5	35.0	1.6	426	5277	rough/very rough	27	0.22	6.0
concrete/grSa	7.7	52.0	2.6	436	12193	very rough	32	0.19	8.9

## Data Availability

Not applicable.

## Appendix A

Explanation of the symbols used in the order of their appearance in the text:

$K_{a}$ = coefficient of active earth pressure [-]

$K_{p}$ = coefficient of passive earth pressure [-]

K = coefficient of earth pressure at rest [-]

$m_{t}$ = auxiliary sum of other parameters [°]

$m_{w}$ = auxiliary sum of other parameters [°]

$\alpha_{a}$ = inclination of failure wedge on the active zone [°]

$\alpha_{p}$ = inclination of failure wedge on the active zone [°]

β = slope angle of the ground behind the wall (upward positive) [°]

$\sigma_{h}^{\prime }$ = effective horizontal stress [Pa]

$\sigma_{z}^{\prime }$ = effective vertical stress [Pa]

δ = friction angle between the retaining wall surface and the soil [°]

r = roughness ratio [-]

τ = the shear stress acting on the structure [Pa]

$\tau_{cr}$ = critical shear stress in the soil mass at failure [Pa]

$\varphi^{\prime }$ = effective friction angle [°]

φ = friction angle [°]

z = vertical distance [m]

*R_inter_* = interface strength reduction factor [-]

v = auxiliary sum of other parameters [°]

γ = unit weight [kN/m^3^]

θ = angle of inclination of the wall to vertical [°]

μ = coefficient of friction [-]

d = distance measured by the laser scanner [m]

$c_{L}$ = speed of light [m/s]

t = time of passage of the electromagnetic wave from the transmitter to the receiver [s]

$\varphi_{d}$ = phase difference between received and sent signal [rad]

T = period of the modulated signal in laser scanners [s]

U = soil graining index [-]

$d_{10}$ = the particle diameters, which together with the smaller ones constitute 10% of the mass of the sample [mm]

$d_{50}$ = the particle diameters, which together with the smaller ones constitute 50% of the mass of the sample [mm]

$d_{60}$ = the particle diameters, which together with the smaller ones constitute 60% of the mass of the sample [mm]

$R_{n}$ = normalized surface roughness [-]

$R_{max}$ = maximum peak to valley height, equivalent of Rz (for profile method) or Sz (for areal method) in code [23] [mm]
